# Structural Basis of Resistant Starch (RS) in Bread: Natural and Commercial Alternatives

**DOI:** 10.3390/foods8070267

**Published:** 2019-07-19

**Authors:** Laura Roman, Mario M. Martinez

**Affiliations:** School of Engineering, University of Guelph, Guelph, ON N1G 2W1, Canada

**Keywords:** high-amylose, digestion, bakery, retrogradation, glycemic response, amylose, amylopectin, α-amylase

## Abstract

Bread is categorized as having a high amount of rapidly digested starch that may result in a rapid increase in postprandial blood glucose and, therefore, poor health outcomes. This is mostly the result of the complete gelatinization that starch undergoes during baking. The inclusion of resistant starch (RS) ingredients in bread formulas is gaining prominence, especially with the current positive health outcomes attributed to RS and the apparition of novel RS ingredients in the market. However, many RS ingredients contain RS structures that do not resist baking and, therefore, are not suitable to result in a meaningful RS increase in the final product. In this review, the structural factors for the resistance to digestion and hydrothermal processing of RS ingredients are reviewed, and the definition of each RS subtype is expanded to account for novel non-digestible structures recently reported. Moreover, the current in vitro digestion methods used to measure RS content are critically discussed with a view of highlighting the importance of having a harmonized method to determine the optimum RS type and inclusion levels for bread-making.

## 1. The Importance of Bread in the Human Diet

Carbohydrates are the most important source of dietary energy for humans (45–70% of total energy intake) [1], with starch being the main structure-building macro-constituent in many foods, including bread, pastry, breakfast cereals, rice, pasta, and snacks. White bread, with an average consumption of about 170 g per day per person in 10 European countries, contributes to the highest proportion of carbohydrates to the daily dietary intake [2]. Despite current findings showing dose-response relation between consumption of whole grains and the risk of non-communicable diseases [3], white wheat bread remains consumers’ first choice mainly owing to its sensory attributes [4]. This event remarkably highlights the technological challenge of the incorporation of dietary fibers to make palatable breads acceptable by consumers, that is, the type and amount of dietary fiber ingredients must be meticulously selected based on their impact on bread quality [5]. 

Besides lacking the nutritional components from the whole grain fraction, white bread is categorized as having a high amount of rapidly digestible starch. This is the result of starch gelatinization produced as a consequence of the high temperatures that the dough reaches during baking (≥70 °C) at relatively high-water content (≥35%) [6,7]. In fact, a complete starch gelatinization in white bread crumb almost always occurs [6,8,9]. In this regard, consumption of white breads, which results in a rapid increase of the postprandial blood glucose, is associated with poor health outcomes including type 2 diabetes, obesity, cardiovascular disease, as well as other metabolic-related health problems [10,11,12].

**Figure 1:**
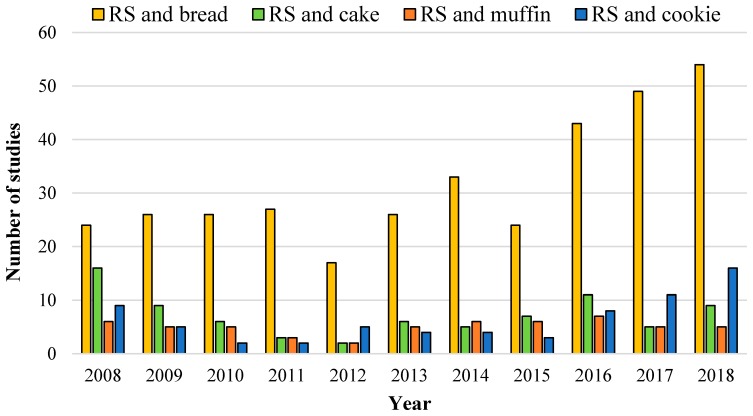
Literature search of the last 10 years on the topics: “resistant starch (RS) and bread”; “resistant starch and cake”; “resistant starch and muffin"; and “resistant starch and cookie”. Data collected from all databases from the Web of Science on 28 June 2019.

In view of the large consumption of daily white bread and the health benefits associated with higher dietary fiber consumption [13], the enrichment of bread crumbs with resistant starch (RS) ingredients is gaining prominence (Figure 1) and can definitively be positioned as an impactful strategy to improve human health through the diet. A literature search in the topic also revealed significantly more studies of RS in breads than in cakes, muffins, and cookies. Because the RS property can change during baking, this review will cover the structural factors responsible for the RS digestion property and the thermal stability of RS ingredients to manufacture breads with meaningful health outcomes. In this review, the structural basis for the RS property of RS in breads will be revised based on recent pivotal studies. Furthermore, the definition of RS will be discussed, addressing holistically and briefly the current analytical methods for quantifying the RS content of foods and the current regulations in terms of food labeling and health claims. We expect that this review provides a brief overlook of the currently commercially available RS ingredients, with special focus on those that support clean and natural labels (i.e., RS4 will not be discussed). 

## 2. RS Definition and Analytical Methods

Resistant starch (RS) is defined as the starch portion that escapes digestion by human enzymes in the upper part of the gastrointestinal tract, entering the large intestine where it can be partially or fully fermented by colonic microflora. The main health outcomes of RS consumption can be categorized mainly based on a modulation on the glycemic response, body weight control, and bowel health. However, this review is not intended, by any means, to provide deep insights into the complex effects of RS consumption on specific metabolic responses and health benefits, which has been previously revised elsewhere [5,14,15,16,17,18,19,20,21,22,23,24,25,26,27].

According to its definition, RS should be predicted by physiological (in vivo) techniques [28], such as the human ileostomy model, where ileal digesta from adults with permanent ileostomies is analyzed for its starch content and compared with the total amount of starch ingested during the study period [16]. However, in vivo methods are remarkably slow and tedious, and require a considerable investment in specialized resources and expertise. Added to that, the rate and extent of starch digestion depends on both extrinsic (e.g., chewing, hormone responses, enzyme activity, passage rate, individual health) and intrinsic (food structure) factors, with the former providing a high variability included in in vivo experiments. On the other hand, the variability from extrinsic factors is excluded in in vitro methods, enabling information for understanding the mechanism of food structural changes during the digestion time course [16]. 

Many in vitro assays for RS determination are variations on Berry’s [29] modification of Englyst’s original method [30]. Starchy products “as eaten” are subjected to gastric (protease) and luminal (pancreatic α-amylase) digestions under fixed physiological conditions of temperature, pH, viscosity, and rate of mechanical mixing similar to those in the gastrointestinal tract. RS is determined by difference between total and digestible starch [31], with validated in vivo results using the ileostomy model [32]. Digestion products are obtained at 20 and 120 min of incubation with α-amylase and further converted to glucose for colorimetric [31] or chromatographic quantification [33]. In the Englyst test, rapid digestible starch (RDS) is the starch digested fraction within the initial 20 min digestion, slowly digestible starch (SDS) is the digested fraction between 20 and 120 min, and RS is the remaining portion after 120 min.

In 2002, McCleary and Monaghan [34] also developed a wide spread method to determine RS, which was validated by both the Association of Official Analytical Chemists [35] (AOAC Method 2002.02) and the American Association of Cereal Chemists [36] (AACC Method 32-40.01). In this case, starchy foods are simultaneously incubated with pancreatic α-amylase and amyloglucosidase for 16 h (vs. 3 h in the Englyst test) in order to hydrolyze and solubilize all the digestible starch. The non-digested starch, the RS fraction, is recovered after several washes and centrifugation steps, and the RS pellet is dissolved with potassium hydroxide prior its hydrolysis to glucose and colorimetric determination. Several other methods were also proposed for analytical determination of RS [37,38,39,40,41].

RS can also be measured following the procedures used for dietary fiber determination. However, attention should be paid on the methodology used because some RS sources can be underestimated. Thus, the Prosky [42] and Lee [43] methods, as well as AOAC official methods 985.29 (AACC 32-05.01) and 991.43 (AACC 32-07.01), respectively, do not quantitatively measure all the RS. Because of the initial heating step at above 90 °C, thermally unstable RS fractions, such as RS2 from banana or potato, are partially degraded. To alleviate this problem, an integrated procedure for the measurement of total dietary fiber (AOAC Methods 2009.01/2011.25; AACC Methods 32-45.01/32-50.01), which fully includes RS (in the same way as in AOAC 2002.02) and other non-digestible oligosaccharides [44,45], was proposed. Therefore, the combination of AOAC 2009.01 and 2002.02 methods could provide quantitative determination of total dietary fiber (including all the RS fractions) and RS, respectively. However, because of the simplicity of AOAC 2002.02, this procedure is recommended if only RS is the dietary fiber of interest.

RS is usually categorized following the RS classification given by Englyst, Kingman, & Cummings [31]; Eerlingen & Delcour [46]; and Brown et al. [47] based on the structural features conferring its resistance. In this way, RS is usually listed into five categories, as follows. RS1: physically entrapped, non-accessible starch in a non-digestible matrix; RS2: native granular resistant starch (B- or C-polymorph); RS3: retrograded starch; RS4: chemically modified resistant starch; and RS5: single amylose helix complexed with lipids. In Table 1, the structural features conferring the RS property within each category (reported to date) are listed and categorized based on the RS classification given by Englyst, Kingman, & Cummings [31]; Eerlingen & Delcour [46]; and Brown et al. [47]. Although this traditional categorization is the most used to date, it is noteworthy that it assumes RS to be a thermodynamically defined structural form (physical entities) and discards its potential kinetic nature. If RS was simply thermodynamically defined, only highly chemically-modified starches (RS4) would be completely resistant to enzyme hydrolysis. This is a critical point in bread-making, as flour/starch fabrication and baking will strongly alter the RS type and content [6,48,49,50]. As an example, baking will generally destroy RS1 and RS2, but may form RS3 and RS5, generally resulting in breads containing RS < 2.5% (dry matter) [40]. In this section, the structural types of RS listed in Table 1 will be briefly described and linked to their effects on bread physical and nutritional quality. Special attention will be put on commercially available RS2 and RS3 clean ingredients (see Section 4 and Table 2). Resistant maltodextrins, soluble chemically modified-dextrins derived from starch and included in the definition of RS, are also commercially available. However, this review will only focus on RS excluding starch degradation products that may also be resistant to digestion by pancreatic α-amylase.

## 3. Natural RS Ingredients in Bread-Making and Structural Basis of Their Resistant Digestion

### 3.1. Physical Barriers Comprising Plant Cell Walls and/or the Food Matrix (RS1)

The resistant digestion property of RS can be the result of its confinement within the intact plant cell (surrounded by the plant cell wall) and/or the food matrix. Overall, the role of cell walls in limiting starch digestion is based on three mechanisms [51,52,53,54,55,56,57]: (1) the difficulty for amylase to permeate through the cell wall; (2) the limitation of starch gelatinization during cooking; and (3) the binding of α-amylase by cellulose and other cell wall components. Whole or partly milled grains or seeds with intact cell walls are clear examples of physically confined starch within cell walls. Milling should be performed carefully to avoid the loss of RS1, as the tissue matrix (cell wall and protein network) could be damaged [57,58]. The effects can be minimized with coarse milling or selection of large particles after mechanical fractionation [57,59]. Nonetheless, large particles are not always suitable and the selection of plant materials with thicker and less permeable cell walls, such as legume flours [52,54] or cereal flours from hard endosperm [57], could increase the content of starch that escapes digestion entirely, even after cooking. 

The presence of whole or partly milled grains and seeds has been reported to decrease the glycemic index of breads [60,61]. However, the use of intact kernels (or broken kernels) will always impact significantly the bread physical and sensory properties. Therefore, food technologists should bear in mind that white bread is the most consumed bread type nowadays [4]. There is little doubt about the health benefits associated with a higher consumption of whole grains [3]. However, to what extent can the particle size of intact grains be reduced to result in breads with lower starch digestion (glycemic response)? Interestingly, Edwards et al. [55] demonstrated that fully cooked and gelatinized porridges, made with 2 mm wheat flour particles, resulted in significantly lower blood glucose, insulin, C-peptide, and glucose-dependent insulinotropic polypeptide concentrations than porridges made with <0.2 mm particles. In fact, they showed that the structural integrity of coarse wheat particles was retained during gastroileal transit using a randomized crossover trial in nine healthy ileostomy participants. However, flours for bread-making are usually smaller than 250 μm and complimentary studies should be performed with smaller variations in particle size. Martinez, Calvino, Rosell, & Gomez [62] observed that among <250 μm flour particles, a differential of 100 μm (coarser) can result in a lower rate and extent of starch digestion, even after full gelatinization through high-shear extrusion. Nevertheless, their effect after incorporation into breads has received little attention. Only de la Hera et al. [63] observed that breads made with coarser rice flour (132–200 μm) presented higher RS than those made with fine flours (<132 μm). On the other hand, Protonotariou, Mandala, and Rosell [64] did not observe differences in the amount of RS between breads made with whole wheat flours with particle size ranging from 57 to 120 μm. Remarkedly, these two studies included the RS values of bread samples after freeze-drying and milling. Even if freeze-dried crumb samples were corrected for moisture and sieved to discard particle size effects, this approach for sample preparation still disregards potential changes in the permeability of the intact plant cell and/or the food matrix. In any case, differences in RS were small and human intervention studies should be performed to confirm or discard the use of coarse flours feasible for bread-making for better postprandial metabolism. Added to that, it should be noted that the amount of ungelatinized starch is dramatically higher in bread crust than in bread crumb [6], and hence the effects of varying particle size could be completely different between crumbs and crusts. In this sense, de la Hera et al. [63] and Protonotariou, Mandala, & Rosell [64] investigated the RS content in bread slices containing the crust portion, so the question of whether particle size differences in the range of 100 μm affect RS in bread crumb, the major fraction of a bread slice, remains unclear. 

Besides plant cell walls, storage proteins from certain plants, such as those from wheat (glutenin and gliadin), maize (zein), and sorghum (kafirin), have the ability to form disulphide bonds that result in a continuous layer around starch granules upon cooking, and in a slowdown of starch digestion [65,66]. In any case, the effect of network-forming proteins on the resulting RS (or glycemic response) after baking has received little attention. Only Berti et al. [67] and Jenkins et al. [68] showed lower postprandial glucose levels of gluten-containing breads compared with gluten-free breads, which was attributed to the presence of a protein network encapsulating the starch. Jenkins et al. [68] also proved that the addition of gluten to gluten-free breads did not reduce the glycemic response, suggesting that the protective effect of the protein present in the wheat is the result of the natural junctions between protein and starch, and is lost once the protein–starch network is disrupted. On the other hand, zein and kafirin, presumably owing to their relative hydrophobicity and disulphide bond cross-linking [69], are isolated in protein bodies in the endosperm cells of the mature grain [70]. The localization of storage proteins in protein bodies, unlike what occurs in wheat, prevents the formation of a continuous matrix around the starch granules within the cells. For zein and kafirin to be functional in doughs, the protein bodies must be disrupted during dough mixing and the proteins freed. However, disruption of the protein bodies has only been observed to occur during high shear extrusion [71] or roller flaking [72].

### 3.2. Granular Surface Properties (Granular Resistant Starch, RS2)

**Figure 2:**
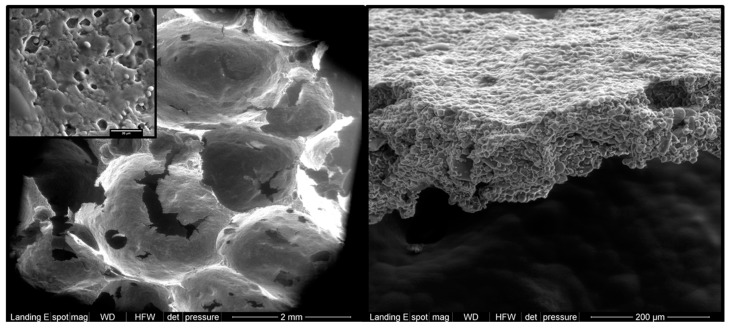
Micrographs of crumb (left) and crust (right) sections of breads containing 20% of RS2 banana starch. Detailed magnification (20 µm) denotes the presence of some granules in the gelatinized crumb.

Starch usually gelatinizes in the range of 54 to 76 °C at ≥20% water [73]. Therefore, considering that, even for those breads made with the lowest possible hydration level (refined dough bread, also known as candeal bread), the moisture content in the crumb is above 35% throughout baking (where a temperature above 70 °C is reached [7]), an extensive (mostly complete) starch gelatinization (Figure 2) is expected to occur [6]. On the contrary, the fast evaporation of water from the crust owing to its high surface temperature impairs the full gelatinization of the starch [6]. In this way, it is possible to find from 56% to 70% (or even higher) of the starch in the crust ungelatinized (Figure 2), depending on the type of bread [6,9]. Restriction of swelling and gelatinization can also be achieved by the interplay of starch with other ingredients in the formula, including lipids, protein, fibers, and sugars [74]. In any case, the presence of starch granules inherently resistant to digestion (RS2) could increase the final content of RS in breads coming from their crust portion [9].

RS2 has been found in ungelatinized tubers, particularly in potato, as well as in starchy fruits, such as green banana, both in vitro [31] and in vivo [32,75]. High-amylose starch is also a source of RS2. High-amylose starch, which is found mainly in maize, is obtained by mutation of the amylose-extender (ae) gene and the gene encoding starch branching-enzyme I [15]. Thus, this starch presents longer branch chains of intermediate material and higher amylose content [76]. RS2 starches are present in starch granules containing the B-type crystalline allomorph. Although differences in the crystalline structure help explain the higher resistance to amylolytic enzymes of potato, high amylose, and banana starches, crystallinity itself does not fully explain the resistance of these starches. At a superior level of starch structure, A-polymorphic starches are reported to have pores (0.1 to 0.3 μm diameter) and channels (0.007 to 0.1 μm diameter) through which α-amylase (around 3 nm radius) could diffuse [77]. On the contrary, larger “blocklets” at the periphery of B-type polymorphic starch granules result in the absence of pores and channels [78], which could significantly limit the enzyme digestion, and possibly be the primary determinants for the RS property [25,79].

In general, the addition of RS2 ingredients may result only in a moderate increase of RS in the final bread, as gelatinization will destroy their semi-crystalline granular structure. This moderate increase will be the result of remaining ungelatinized granules in the crust, which represents a significant, but lower portion of the bread slice. As an example, Roman, Gomez, et al. [9] observed an RS increase from 0.26 to 5.66% in the crust with the replacement of the main starchy ingredient by native banana starch, but no significant RS increase was observed in the crumb portion. On the contrary, native high amylose is the only RS2 source that resists gelatinization, making this starch more suitable for hydrothermally-processed foods. In fact, complete gelatinization of these mutant starches is only achieved at temperatures higher than 120 °C [5,80,81]. In addition, once gelatinized, high amylose starches can form high amounts of RS3 [82]. Thus, several types of resistant starch, namely, RS2, RS3, and RS5, can coexist in the final bread.

### 3.3. Dispersed Starch Molecules Forming Resistant Starch upon Cooling and Storage (RS3)

After gelatinization, which results from baking, dispersed starch molecules begin to re-associate upon cooling, forming tightly packed structures stabilized by hydrogen bonding that are more resistant to digestion [83]. The resistance of retrograded amylose to α-amylase digestion was demonstrated both in vitro and in vivo long ago [84], which was termed as RS3. The amount of RS3 produced from retrograded amylose is dependent on the amylose ratio and its chain length [18,85]. Similar to RS2, the enzyme resistance of RS3 has been associated with the formation of a highly thermostable B-type crystalline structure. Thus, the increased crystallinity is expected to result in fewer available α-glucan chains to which α-amylase can bind and thus reduce the susceptibility of retrograded starch to digestion [82]. Nonetheless, crystallinity itself does not fully explain the resistance of RS3, as previously mentioned with RS2. Amorphous material in enzyme-resistant fractions has been found, confirming that the resistance is not simply based on a specific crystalline structure that is fully undigested [86]. Cairns et al. [87] and Gidley et al. [88] suggested that the resistance to digestion is also the result of other double helices not involved in crystals. More recently, extrusion processing of high amylose starch was shown to result in non-crystalline dense packing of amylose chains upon cooling, which exhibited significantly higher RS content than the cooked counterpart [82,89,90]. Furthermore, the content of RS in extruded high amylose starch was similar to that in a granular native state [82]. We believe that the increase in amorphous RS during extrusion could be the result of the molecular fragmentation of amylose and amylopectin chains during extrusion, which could improve molecular mobility and amorphous molecular packing at submicron length scale. In fact, recently, evidence of shear-induced amylose scission during extrusion has been reported [91]. 

In contrast to amylose, the branched structure of amylopectin is less prone to retrograde, needing a longer time for the formation of double helical structures [91]. Retrograded amylopectin has been linked to the formation of slowly digestible starch (SDS), and hence to a reduction in the rate of starch digestion [92,93]. Starch with a slow digestion rate has been proposed to partially pass to the large intestine as RS, where it functions as a source to bacterial fermentation [84]. In this way, although RS3 has been generally attributed to the formation of resistant crystalline structures from amylose double helices, some old and recent evidence suggests that retrograded amylopectin should be included as another form of RS3 [84]. In fact, Englyst and Macfarlane [94] already proposed a further classification of RS3 into two subcategories, that is, RS3a and RS3b, comprising retrograded amylopectin and amylose, respectively. In terms of amylopectin, slowly digestible starch structures involving amylopectin have been attributed to the following: (1) high proportions of long chains [93,95]; (2) chains with longer average length [9,92,93]; and/or (3) lower molecular sizes through processes such as acid-hydrolysis or high shear cooking extrusion [9,91,93]. In contrast to RS3 from amylose retrogradation, which is thermally stable (melting of amylose-amylose double helices occurring at ~150–160 °C), double helices or aggregates of double helices involving amylopectin melt at significantly lower temperature (~55 °C) [9,96,97] and, therefore, attention should be paid when using in breads that will be re-heated.

### 3.4. Introduction of Chemical Structures (Chemically Modified Resistant Starch, RS4)

Starch resistance can also be created by the inclusion of chemical structures along starch chains. The resistance to digestion of chemically modified resistant starch (RS4) is dependent on the type and extent of the chemical modification, mostly consisting of dextrinization, etherification, esterification, oxidation, and/or cross-linking [58].

The mechanisms responsible for the enzymatic resistance of RS4 have been revised elsewhere [98]. It is originated principally by two different mechanisms: (1) the introduction of bulky functional groups (e.g., oxidation, etherification, or esterification with hydroxypropyl, acetyl, and octenyl succinic anhydride groups, among others) and (2) starch cross-linking (typically with phosphate groups). In the former category, large and bulky side functional groups are added by substitution along the α-1,4 D-glucan chains to hinder the enzymatic attack, which also makes adjacent glycosidic bonds inaccessible to the enzymes. As for the latter, the presence of cross-linked starch chains (reaction with two or more hydroxyl groups) inhibits granular swelling, preserves granular integrity (preventing enzyme access), and creates steric hindrance, making amylase unable to properly bind to starch. Furthermore, some of the abovementioned chemical modifications can bring about RS ingredients with up to 68–79% RS. Nonetheless, these chemical methods are characterized by long reaction times (up to 24 h) and environmental concerns (use of excess reagents that need to be properly removed and disposed of). Therefore, this type of modification seems less appropriate nowadays in view of the current health and wellness megatrends, which are orientated to clean and natural (free of chemicals) labels.

RS4 from different starch sources are a widely commercialized RS ingredient, although little information exists from the manufacturer about the nature and level of these modifications. Multinational companies providing RS4 from potato, tapioca, wheat, and/or high-amylose maize include Ingredion [99], Roquette [100], MGP Ingredients [101], and Cargill [102]. Ingredion provides RS4 from high-amylose maize starch and potato starch known as Versafibe 2470 and 1490, respectively. Roquette offers a line of modified starches under the name “CLEARGUM” comprising acetylated, diphosphate, and octenyl succinic anhydride (OSA) starches. MGP ingredients offers a phosphorylated cross-linked starch under Fibersym brand name. Cargill also offers a range of stabilized RS4 starches (C☆PolarTex, C☆StabiTex, C☆Tex), subjected to different chemical modifications (hydroxypropylated, acetylated, phosphorylated starch, and so on). Several research works have focused on the influence of these chemically modified RS starches in RS content, glycemic index, and quality of breads [103,104,105,106,107,108,109,110,111]. Chemically modified starches preserve their RS property during conventional food hydrothermal processing and, therefore, can significantly increase the RS content in bread. Nonetheless, based on consumers’ demands for clean labeled products, these investigations will not be discussed in the present review.

### 3.5. Lipid Complexed Resistant Starch (RS5)

Amylose can form inclusion complexes with lipids, and these complexes have been shown to be more resistant to digestion [112]. Amylose–lipid complexes naturally exist in some starch sources (principally high amylose starches) [113]. Nonetheless, they can also be formed upon hydrothermal treatments, such as baking, in the presence of exogenous or endogenous lipids (monoglycerides, fatty acids, lysophospholipids, and surfactants) [114]. The stability and resistance to digestion is also dependent on the type of lipid (i.e., carbon unit length and unsaturation) complexed [114,115,116]. 

Two forms of complexes can be distinguished depending on their thermostability: Type I amylose–lipid complexes that melt at about 95–105 °C (less ordered structures), and Type II (V-type crystalline structures) melting at about 110–120 °C [117,118]. 

Although there are no commercially available sources of RS5 in the market, amylose–lipid complexes can also reform upon baking, provided there are lipids in the formula. In this regard, most gluten-free breads, which are mostly made with maize starch and rice flour, incorporate some source of lipid/fat in the formula, to enhance the crumb softness and juiciness, as it tends to be excessively dry [119]. Meanwhile, wheat flour lipids represent 2.0% to 2.5% of the flour and exogenous lipids are often added to reduce hardness or staling [120]. Therefore, the presence of a certain amount of RS5 in breads is expected.

It is worth noting the ~50% reduction of postprandial blood glucose and insulin levels of breads containing 60% (flour basis) of a developed RS5 containing ingredient compared with the control white bread [121]. These authors produced an ingredient containing both RS3 and RS5 by debranching high amylose VII maize starch with isoamylase followed by complexation with palmitic acid. Interestingly, they showed that the debranching treatment increased the amount of linear chains, which could either retrograde or form complex with lipids more effectively (RS: 52.7%) than the native high-amylose starch molecules (RS: 35.4%) upon baking.

## 4. Effects of RS in Physicochemical Characteristics of the Breads

Although bread-making varies widely around the world, the four basic ingredients are flour/starch (normally from cereals and tubers), water, yeast, and salt. Processing conditions include kneading, proofing, and baking. Inclusion of RS ingredients in the formula is usually given by replacement levels of the starchy material by the RS ingredient. Most investigations approached the RS enrichment of breads using commercial RS2 and RS3 ingredients, but only some studies included the RS content in the final product. This is critical as baking will critically alter the type and amount of RS. For this reason, in Table 2, only those studies in which RS was assessed in the final product were included.

High amylose starches, usually from maize, in both granular (RS2) and retrograded (RS3) form, are among the most used commercial RS ingredients in bread-making. They are widely available from many companies including Ingredion, Roquette, Cerestar, and SunOpta Ingredients. Tapioca rich in retrograded amylose (RS3) has also been investigated, which can be purchased from Cargill. RS2 from green banana starch or flour has also been evaluated in bread. The demand for banana starch/flour (RS2) is on the rise, and companies like Chiquita (Costa Rica), Livekuna (Canada), International Agriculture Group (United States), and Natural Evolution (Australia) commercialize a wide range of banana starch/flour ingredients with elevated RS content (~40–50%). On the one hand, it must be brought into attention that, converse to RS2 from high-amylose maize and RS3, RS2 from green bananas is not heat-stable and will not resist baking, that is, there will be a significant fraction of RS that will be lost during baking [9,122]. It is noteworthy that banana RS2 decreases with ripening owing to its conversion into reducing sugars by endogenous α-amylase [123]. This enzyme has been reported to present an optimal activity between 8 °C and 38 °C, starting to be denatured at 38 °C and being fully denatured after 5 min at 100 °C [124]. Therefore, the drying step will be critical for its inactivation and the preservation of RS2 in banana flours. Specifically, Pico et al. [125] showed how oven-dried banana flours at 40 °C for 24 h exhibited an insoluble dietary fiber content of 26.8%, which was significantly lower than the same flours obtained through freeze-drying (43.3%). On the other hand, albeit banana RS is lost during baking, banana starch has been reported to have a suitable molecular structure to result in structurally-driven slowly digestible starch in bread crumb after baking through retrogradation [9], part of which could reach the colon as RS3 (RS3b). This occurrence has been reported to improve through shear-induced fragmentation of amylopectin molecules through high shear extrusion [91], which was attributed by the authors to smaller amylopectin fragments being more mobile and more prone to interact through retrogradation. 

The targeted amount of RS ingredient during formulation depends on the starch being used and the desired RS level in the bread. Normally, percentages of replacement have been reported within 5–30%, which resulted in breads with final RS content being dependent on the method of analysis. The RS content in both ingredients and breads was, in some cases, quantified by the AOAC Method 2002.02 and modifications of the Englyst procedure. However, AOAC official methods 985.29 and 991.43 were also used, which can lead to underestimations of the RS content (Section 2). Therefore, it is unfortunate that the RS content of different RS ingredients and breads is not comparable nor harmonized. It is expected that the CODEX definition of dietary fiber [126], and its adaptation by many worldwide authorities, brings about a unique method of analysis whose adoption enables harmonized information about the RS content in different commercial RS ingredients and RS-containing foods. This would also answer existing uncertainties in the association of RS consumption through breads with positive health outcomes. It is very important to mention that the clear majority of studies did not report information about the day RS was analyzed (i.e. time after baking), which masks information about the structural basis of the RS in breads, especially of those containing RS3, which increases over time through retrogradation [48,93,127,128,129]. Another masking factor is the fact that the nature of the sample for RS analysis is unknown and not reported in most studies, that is, whether crumb, crust, or the whole slice was analyzed (Table 2). This is particularly important considering the differences in the degree of gelatinization between crumb and crust (Figure 2) [6]. 

The incorporation of RS ingredients into bread formula also brings about differences in the physicochemical and organoleptic properties of the bread (Table 2). The flavor, mouth-feel, appearance, and texture are examples of important quality factors to bear in mind for good consumers’ acceptance. As reported in Table 2, formulation of breads with increasing levels of RS2 and RS3 sources, in general, has detrimental effect on volume, hardness, cohesiveness, and crust color. On the basis of Table 2, approximately a 20% replacement of wheat flour by RS ingredients seems to be adequate to keep bread final quality, although lower specific volume and harder and/or less cohesive crumbs were generally observed. Paler crusts are visible in some studies owing to the whitish color of starch and the reduction in protein content available for Maillard reaction, while the color of the crumb seems less affected. Meanwhile, the incorporation of banana flour led to both darker crumb and crust [9]. Differences in crumb cell size distribution and decreased gluten network formation have also been reported [127,130,131,132] On the other hand, consumers’ perception generally reflected similar or unaffected sensory evaluation, which may be because of the bland flavor of most RS sources. In this regard, Almeida et al. [133] studied the effects of adding different dietary fiber sources and concluded that RS2 (high amylose maize) was a more “inert” fiber source in relation to bread quality characteristics. RS2 was found to have lower water holding capacity than other dietary fibers, and thus less impact on dough rheology, resulting in breads with superior quality [134].

In addition, other than the nature of RS ingredients, processing conditions may also influence the formation of RS in bread. Baking under low-temperature and a long-time period significantly resulted in higher amounts of RS in bread than in those baked under higher-temperature and shorter time [48,49,135,136]. Similarly, higher addition of water in the formula has also been reported to increase RS in the bread. The higher the water content in the dough, the more starch can be gelatinized, resulting in increased starch retrogradation (RS3) during cooling of gelatinized starch [136]. RS3 in wheat bread has been reported to be greater for refrigeration than ambient or frozen temperatures [129], so for certain starches, refrigeration temperatures may boost their RS property in breads. 

In some studies, the amount of water added to each formulation was adjusted based on the water binding capacity of starches as determined by farinographic analysis or elastic modulus [127,130,131,137,138,139]. In general, these RS rich ingredients have higher water absorption capacity than wheat flour or gluten-free flours used for bread making, especially if RS3, in non-granular form, is used [130,139]. Therefore, if water content is not properly adjusted, especially in gluten-containing breads, higher water absorption by RS ingredients in the dough can result in detriment of the gluten network [131,140]. Low water availability causes non-optimal repartition of water among dough components and may lead to final breads with detrimental quality characteristics in terms of specific volume, textural attributes, and appearance [141]. It is important to highlight that despite water adjustment in the formula, the specific volume always decreased when high levels of RS ingredients were added into the formulation of gluten-containing breads. This could be explained by the extent of gluten protein dilution [142] and a hindrance effect on the gluten network development by the non-gelatinized high maize starch granules [130]. Conversely, in gluten-free breads, no differences or even an improvement in bread volume with RS inclusion were observed in some studies [9,136,143].

## 5. Current Legislation of RS Ingredients and Products in the Food Industry

RS generally meets the criteria to be defined as “dietary fiber” by the comprehensive dietary fiber definition adopted by the CODEX Alimentarius Commission [126]. However, isolated or synthetic RS ingredients require the American Food and Drug Administration (FDA) [144] or European Union (EU) approval [145] after assessments of scientific evidence relating RS to physiological benefits. Under that proposed outline, “isolated” (pure RS2) or “synthetic” (RS3, RS4 and RS5) RS sources would remain outside this definition. Nonetheless, in June 2018, the FDA [144] released a review of the scientific evidence on the physiological effects of certain non-digestible carbohydrates, which decided to include isolated RS2 ingredients, such as raw green banana, potato, and high amylose starches, in the definition of dietary fiber. According to this categorization, in Europe (2 kcal/g), Australia (2 kcal/g), and USA (0 kcal/g), RS has a lower energy value compared with other non-fiber carbohydrates (4 kcal/g) [23,146]. The European Commission [147] also allows manufactures to voluntarily claim foods as a “source of fiber” if it contains at least 3 g of fiber per 100 g, and as “high in fiber” if it contains at least 6 g of fiber per 100 g. 

Current regulations also identify the potential physiological benefits of RS. The European Food and Safety Authority (EFSA) approved the health claim, “Replacing digestible starch with resistant starch induces a lower blood glucose rise after a meal”. However, this claim can be only used when the final RS content in the food is at least 14% of the total starch [148]. On the other hand, from 2016, the FDA [144] has allowed manufacturers to use the claim related to high amylose maize RS, “High-amylose maize resistant starch, a type of fiber, may reduce the risk of type 2 diabetes, although FDA has concluded that there is limited scientific evidence for this claim”. So far, to the best of our knowledge, there is no other RS source with an authorized health claim in the United States.

## 6. Conclusions

The development of breads rich in RS and acceptable quality attributes could have a positive impact on the modulation of the glycemic response, the control of body weight, and the improvement of bowel health of bread consumers. The growing evidence of the positive health outcomes attributed to RS is leading to the apparition of novel RS ingredients in the market for bread-making, whose incorporation may seem the most logical and easy strategy to increase the RS content in breads. However, it must be noted that not all RS ingredients preserve the RS property during baking. It is thus paramount to understand the structural basis for their resistance to digestion and hydrothermal processing, which is often disregarded. This review concludes that high amylose starches, both native (RS2) and processed (RS3), are the most suitable RS ingredients for bread making in terms of RS preservation during baking and a lower detrimental impact on bread texture. However, their level of inclusion must be carefully selected. Another issue that this review addresses is the lack of harmony in RS values, which is the result of using different in vitro methods, some of which do not account for all types of RS structures. This outlook is changing though, as AOAC Method 2002.02 or any of its extensions, such as AOAC Methods 2009.01/2011.25, are adopted by many researchers from different nationalities.

## Figures and Tables

**Table 1:** Structural features conferring the resistant digestion property within each clean-label resistant starch (RS) category.

Classification	Structural Features Conferring the RS Property within Each Category	Detrimental Steps That May Decrease RS Content during Bread-Making	Assisting Steps That May Increase RS Content during Bread-Making
**RS1**	Intact plant tissues	Milling, sieving, baking	-
	Highly dense food matrices	-	Baking and cooling
	Confined starch within a continuous layer of certain proteins	-	Baking of starch materials containing specific layer forming proteins
**RS2**	Starch granules with an outer high-density shell structure	Baking (of note that high amylose RS2 is more heat-resistant)	-
**RS3**	Retrograded amylose	-	Baking and cooling
	High-density processed amylose	-	Extrusion of high amylose starch ingredients
	Retrograded amylopectin	Baking	Baking and cooling
**RS4**	Chemically substituted starches	-	-
	Chemically cross-linked starches	-	-
	^a^ Resistant maltodextrins	-	-
**RS5**	Amorphous amylose-lipid complexes (form I)	-	Baking and cooling
	Crystalline amylose-lipid complexes (form II)	-	Baking and cooling

^a^ Resistant maltodextrins can be defined as chemically-modified dextrins instead of chemically-modified starch. In that case, they should be excluded from this list.

**Table 2:** In vitro studies on commercially available RS2 and RS3 sources as ingredients to increase RS content in wheat- and gluten-free breads.

Ingredient	RS Content	Type of Bread	Substitution Level (%)	Evaluation Day	RS Content	In vitro RS Method	Effects on Bread Quality	Refs.
HA maize starch, Hi-Maize 260, Ingredion	60% IDF Manufacturer (58.4% TDF)	Wheat flour (4.5% TDF)	0 10 20 30	n.a. crumb	6.6% bb, db 9.5% bb, db 17.0% bb, db 26.6% bb, db	AOAC 985.29	Increased hardness Decreased cohesiveness and resilience Decreased volume with 30% level Lighter crust Decreased number of cells in the crumb Decreased C_inf_ and estimated GI Increased consumer acceptability (20% level)	[130]
HA maize starch, Hi-Maize 260, Ingredion	60% RS Manufacturer	Wheat flour	0 10 15 20	2 h, Lyophilized crumb	1.2% n.a. 3.9% n.a. 5.9% n.a. 11.1% n.a.	Goni et al. [40]	Decreased volume Increased hardness with 15% level Lighter crust Staling dependent on the level of replacement	[142]
HA, Amylo-maize starch N-400, Roquette	40% TDF Manufacturer	GF flour mix (maize starch, rice flour, tapioca starch)	0 20—RS2 20—RS3 ^a^	n.a. bread	1.2% bb, db 4.4% bb, db 7.6–9.2% bb, db	Englyst et al. [31]	Reduced in vitro glycemic index (RS3 > RS2)	[137]
HA maize starch, Hi-Maize 260, Ingredion	56% TDF Manufacturer	Yellow maize flour	0 20	n.a.	4.3% bb, db 12.0% bb, db	Modified AOAC 2002.02	Specific volume and texture were not modified Decreased cell density Similar sensory evaluation SDS fraction was also increased eGI decreased from 85 to 71	[143]
HA maize starch, Hi-Maize 260, Ingredion	56% TDF Manufacturer	White maize flour	0 20	n.a.	5.5% bb, db 11.3% bb, db	Modified AOAC 2002.02	Specific volume and texture were not modified Same cell density Similar sensory evaluation SDS fraction was also increased eGI decreased from 83 to 72	[143]
HA maize starch, Eurylon, Roquette	83.2% RS2	Wheat flour (14.1% RS)	0 20 ^b^	24 h/7 days, Lyophilized crumb	0.0%/4.4% bb, db 7.7%/10.2% bb, db	Modified Englyst et al. [31]	Decreased specific volume Decreased hardness No sensory differences	[127]
Extruded retrograded HA maize starch, EURESTA, Cerestar	29.5% RS3	Wheat flour (14.1% RS)	0 20 ^b^	24 h/7 days, Lyophilized crumb	0.0%/4.4% bb, db 8.4%/11.0% bb, db	Modified Englyst et al. [31]	Decreased specific volume Decreased hardness No sensory differences	[127]
HA maize starch, HylonVII, Ingredion	53% RS2	Wheat flour	0 10 20 30	24 h, Lyophilized crumb	1.2% bb, db 4.1% bb, db 8.1% bb, db 10.1% bb, db	AOAC 2002.02	Decreased volume for 30% level Increased hardness for 30% level Paler crust color for 20% and 30% levels	[139]
HA maize starch, Novelose330, Ingredion	46.5% RS3	Wheat flour	0 10 20 30	24 h, Lyophilized crumb	1.2% bb, db 4.7% bb, db 9.7% bb, db 12.7% bb, db	AOAC 2002.02	Decreased volume 20% and 30% levels Increased hardness for 20% and 30% levels Paler crust color for 30%	[139]
HA maize starch CrystaLean, SunOpta ingredients	45% RS3	Wheat flour	0 10 20 30	24 h, Lyophilized crumb	1.2% bb, db 4.4% bb, db 8.3% bb, db 12.4% bb, db	AOAC 2002.02	Decreased volume for 30% level Increased hardness for 30% Paler crust color above 30%	[139]
HA maize starch, Hi-Maize 260, Ingredion	RS > 60% Manufacturer	Maize starch Potato starch (4:1) ^c^	0 10 15 20	n.a.	2.1% bb 3.9% bb 4.7% bb 5.0% bb	AOAC 991.43	Decreased volume Similar initial hardness (20% level) Reduced hardening (48 h)	[138]
Tapioca starch, ActiStar 11700, Cargill	RS3 > 50% Manufacturer	Maize starch: Potato starch (4:1 mixture) ^d^	0 10 15 20	n.a.	2.1% bb 2.5% bb 2.8% bb 3.0% bb	AOAC 991.43	Decreased volume Reduced initial hardness (20% level) Reduced hardening (48 h)	[138]
HA wheat flour, Okumoto Flour milling	6.7% TDF	Wheat flour (3.4% TDF)	0 10 30 50	2h	0.9% bb, db 1.6% bb, db 2.6% bb, db 3.0% bb, db	AOAC 985.29 ^e^	Decreased volume Increased hardness Similar staling (hardness) for 30 and 50% levels Higher staling for 10% level Increasing RS with storage and substitution	[132]
Green banana starch, Natural Evolution	42.2% RS2	Maize starch (0.8% RS): Rice flour (0.1% RS) (1:1 mixture)	0 20—Native 20—Extruded ^f^	24 h, Crumb and crust	1.5% cb – 0.3% ct, db 1.7% cb –5.7% ct, db 1.9% cb –0.7% ct, db	AOAC 2002.02	Darker bread color Improved volume, reduced hardness, and improved sensory acceptance (native banana) Increased SDS fraction in crumb with native and extruded banana	[9]
Green plantain flour, Chiquita	50.1% RS2	Rice flour: GF wheat starch (1:1 mixture)	0 35	24 h	1.1% bb, db 2.3% bb, db	AOAC 2002.02	Improved volume but increased firmness Darker bread crumb Lighter bread crust Optimization of water content, baking time and temperature for 30% replacement to maximize RS (3%)	[136]

SDS = slowly digestible starch; AOAC = Association of Official Analytical Chemists; IDF = insoluble dietary fiber; TDF = total dietary fiber; GI = glycemic index; GF = gluten-free; n.a. = not available; HA = high amylose; bb = bread basis; cb = crumb basis; ct = crust basis; db = dry basis. ^a^ RS3 was prepared with RS from HA maize starch (Amylomaize, N400) subjected to debranching and/or three autoclaving-cooling cycles. ^b^ Wheat flour (24%) was replaced by 20% of RS2 or RS3 source and 4% gluten. ^c^ Maize starch was replaced by HA maize starch. Water level was not modified in the formula. ^d^ Potato starch was replaced by tapioca starch. Water level was increased in the formula. ^e^ TDF and RS in breads after baking and during storage were determined using the total dietary fiber assay kit. The RS in bread was calculated as the amount of non-digestible carbohydrate minus DF that already existed in the flours. ^f^ Banana starch (Native RS2) was extruded under high-shear extrusion to obtain pregelatinized starch (RS3).
